# Dewitt Co. Medical Society

**Published:** 1877-07

**Authors:** W. A. Tyree

**Affiliations:** Secretary, pro tem.; Clinton, Ill.


### DEWITT CO. MEDICAL SOCIETY.

At the annual meeting of the Dewitt Co. Medical Society, the following preamble and resolutions were adopted, after a fair discussion for and against, by a vote 8 to 2:

Whereas, It has been clearly defined by the Judicial Council of the American Medical Association, which is the supreme medical court of the land—(See page 32, vol. 25, and page 41, vol. 20, transactions), that it is a violation of the Code of Ethics to bid or contract for the practice of corporations, county alms-houses, orphan asylums, etc.; therefore, be it

*Resolved*, By the Dewitt County Medical Society, that hereafter it will be considered a violation of the Code of Ethics of the American Medical Association for any member of this society to bid for the pauper practice in the poor-house, or the poor practice in the towns of this county.

*Resolved*, That hereafter it shall be considered a violation of the Code of Ethics, for any member of this society to enter into a contract with the Board of Supervisors to do the pauper practice or the poor practice of this county as an award of competitive bidding.

*Resolved*, That hereafter it shall be considered a violation of the Code of Ethics for any member of this society to enter into a contract to do medical, surgical or other practice for any corporate bodies, unless such bodies fix a reasonable salary for such services.

*Resolved*, That we appeal to the Board of Supervisors of this county, and earnestly request them to fix a reasonable salary for medical and surgical attendance of the paupers in the poor-house, and urge them to procure the best medical and surgical skill that the county affords, that will do the work for the salary.

*Resolved*, That it is the expression of this society, that such a course by the Board of Supervisors would be to the best interest of the poor, and a great saving of money to the county, and would be alike honorable to supervisors and the doctors, and would forever settle the dispute between them.

*Resolved*, That any member of this society, that hereafter bids for the pauper practice of this county, or contracts to do such practice, upon any other terms than are herein specified, shall be declared guilty of violating the Code of Ethics of the American Medical Association, and the rules and regulations of this society, and on proof of guilt shall be expelled from the society, or supended for such length of time as the society may deem proper.

*Resolved*, That the foregoing preamble and resolutions be spread on the records of the society, and published in the Chicago Medical Journal and Examiner, the *Clinic*, of Cincinnati, and the newspapers of this county, and a copy sent to the Board of Supervisors.

Clinton, Ill., April 10th, 1877.

W. A. Tyree,
Secretary, *pro tem.*

---

